# Stress-induced phase-alteration in solution processed indium selenide thin films during annealing†

**DOI:** 10.1039/d1ra01403j

**Published:** 2021-04-13

**Authors:** Bipanko Kumar Mondal, Shaikh Khaled Mostaque, Md. Ariful Islam, Jaker Hossain

**Affiliations:** Solar Energy Laboratory, Department of Electrical and Electronic Engineering, University of Rajshahi Rajshahi 6205 Bangladesh jak_apee@ru.ac.bd; Department of Physics, Rajshahi University of Engineering and Technology Rajshahi 6204 Bangladesh

## Abstract

This article demonstrates the successful synthesis of indium selenide thin films by a spin coating method in air using thiol-amine cosolvents. The synthesized films encountered a transformation from β-In_3_Se_2_ to γ-In_2_Se_3_ phase due to mechanical stress during annealing as confirmed from XRD and EDS analysis. The SEM study ensured the homogeneity and uniformity of surface morphology of both phases. The FTIR analysis also confirmed the In–Se stretching vibration bond for both β-In_3_Se_2_ and γ-In_2_Se_3_ thin films. The temperature dependent electrical conductivity indicated the semiconducting nature of both phases. The optical transmittance was found to increase with annealing temperatures for both phases. The optical band gaps were estimated to be in the range of 2.60–2.75 and 2.12–2.28 eV for β-In_3_Se_2_ and γ-In_2_Se_3_ phases, respectively consistent with the reported values. These results indicate that stress-induced phase transformation in solution-processed indium selenide could be useful in 2D optoelectronic devices in future.

## Abstract 2

This article demonstrates the successful synthesis of indium selenide thin films by a spin coating method in air using thiol-amine cosolvents.

## Graphical abstract

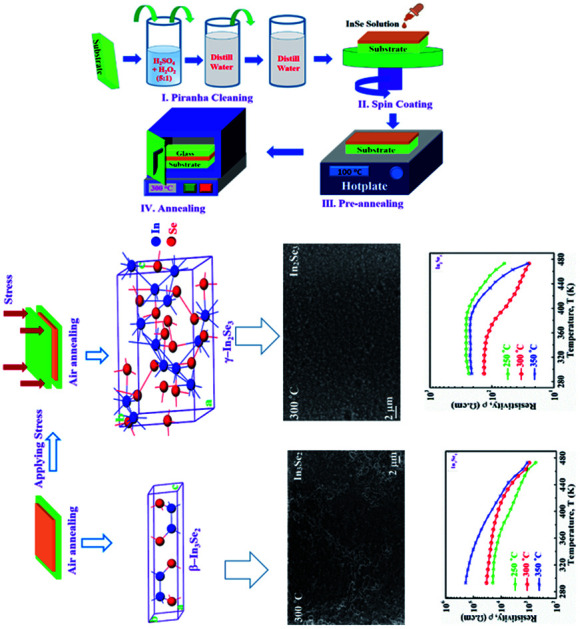

## 1. Introduction

Recently, indium selenide (InSe) has been extensively studied because of its high photo-response, tunable band-gap, phase-transition phenomena and excellent electrical transport properties which have made it available in varieties of potential applications. These unique properties make it a suitable candidate for applications in phase-change memories devices and solar energy conversion.^1–3^ Indium selenide (InSe) belongs to the group of III–VI metal chalcogenide materials. The phase diagram of InSe shows that there are various stable phases with many stoichiometries such as InSe, In_2_Se, In_2_Se_3_, In_3_Se_2_, In_5_Se_6_, In_4_Se_3_, In_6_Se_7_, In_3_Se_4_ and In_2.5_Se_4_ as revealed by many researchers.^4–9^ Among all of the phases, In_2_Se_3_ belongs to α, β, γ, δ, and κ crystalline phases as trivalent and divalent atoms can be amalgamated to fulfill their bonding in the same stoichiometric ratio under different temperatures.^10^ As a phase change materials, In_2_Se_3_ is more fascinated compared to others (for example GeTe, Ge_2_Sb_2_Te_5_ alloy) materials due to renewed optical and electronic properties with the application in multilevel based memories, optoelectronic detectors, sensors and two dimensional (2D) based nano devices.^11,12^

The formation of the In_2_Se_3_ phase is difficult to prepare because it is stable at generally under high pressure and temperature. The phase transformation of β-InSe to γ-In_2_Se_3_ have been also happened above at 200 °C in thermal annealing process.^12^ In our previous study, we have also reported phase transition from In_2_Se_3_ to highly degenerate In_3_Se_4_ phase due to heat treatment.^4,13^ Apart from the temperature, pressure induced phase transition in In_2_Se_3_ phase has also been studied and reported recently.^14^

The different phases of indium selenide thin films have already been synthesized using various deposition methods, for example Hydrothermal,^10^ E-beam evaporation,^15^ Sol–gel,^16^ Spray pyrolysis,^17^ Chemical Vapor Deposition (CVD),^18^ Flash evaporation,^19^ Molecular Beam Epitaxy (MBE),^20^ Van der wall epitaxy,^21^ Thermal evaporation,^22^ Electro deposition^23^ Laser irradiation^24^*etc.* However, among all of the deposition methods, solution processed spin coating is the cheapest and easiest method to deposit uniform thin films which, to the best of our knowledge, is not employed yet to deposit indium selenide thin films. It is already reported that a simple thiol-amine solvent system has the ability to dissolve bulk V_2_VI_3_ (V = As, Bi, Sb; VI = Te, Se, S) semiconductors and chalcogenide materials.^25,26^ The solubility of In_2_Se_3_ within thiol-amine co-solvents is also reported.^27^ However, there is no report of fabrication and characterization of indium selenide thin films by this process.

In this article, we demonstrate the synthesis of InSe thin films in air by spin coating method using ethylene-di-amine and 1,2 ethanedi-thiol co-solvents at various annealing temperatures. We also studied the effect of applying mechanical-stress in the indium selenide thin films during annealing.

## 2. Experimental details

### 2.1 Preparation of solution

**Fig. 1:**
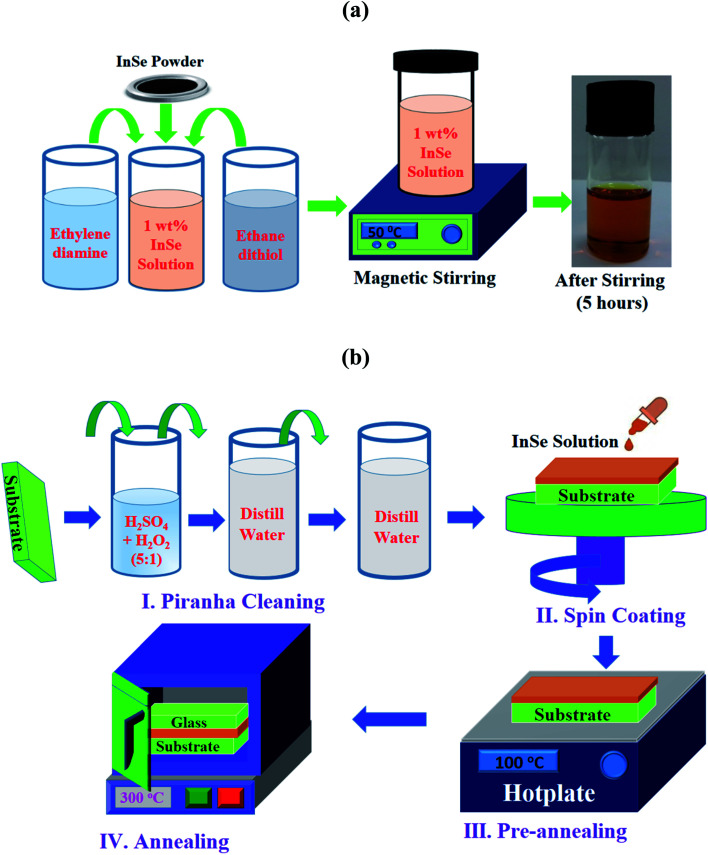
(a) The preparation steps of 1 wt% indium selenide precursor solution and (b) schematic presentation of substrate cleaning and film deposition steps.

To prepare indium selenide solution, InSe granular powder with 99.999% purity was purchased from Metron, USA and co-solvents ethylene-di-amine and 1,2 ethanedi-thiol were purchased from Sigma Aldrich. The preparation steps of indium selenide solution was represented in Fig. 1a. First, 1 wt% InSe powder was dissolved in the diluted co-solvents ethylene-di-amine and 1,2 ethanedi-thiol at a volume ratio 9 : 1. To completely liquefy InSe powder, this solution was stirred using magnetic stirrer at a speed of 350 rpm at 50 °C temperature for 5 hours. After completely dissolving the InSe powder, the solution was light brown in color. Then the solution was filtered using 0.45 μm pore sized syringe filter to remove any additional contaminants from solution.

### 2.2 Deposition of indium selenide thin films

Glass substrates (2 × 2 cm^2^) were used to fabricate indium selenide thin films utilizing thiol-amine cosolvents solution. First, the substrates were cleaned utilizing piranha solution to eliminate gross contaminations and then they were washed by distilled water twice for removing any additional contaminations. After that prepared solution was spin coated on cleaned glass substrate only one time at a speed of 1000 rpm for 30 seconds with a 10 seconds slope at 500 rpm. Then, the deposited indium selenide films were pre-annealed on a hot plate at 100 °C for 10 minutes to dispel residual solvents. After that these pre-annealed films were post-annealed in two different ways for further crystallization of the films. In a first recipe, these films were annealed at 250, 300 and 350 °C temperatures for 10 minutes in a carbolite oven in air without applying any stress. In a second recipe, these film were annealed in the same condition with applying a simple mechanical stress. The mechanical stress was applied on indium selenide thin films using a microscopic glass of 1–1.2 mm thick during annealing. The films were sandwiched between two microscopic glasses which were bound with stainless steel clip on each edges of the glass substrate. The schematic of the substrate cleaning, deposition steps of indium selenide thin films are shown in Fig. 1b.

### 2.3 Characterization of indium selenide thin films

The thickness of the synthesized indium selenide thin films annealed at different temperatures were measured using Bruker Dektak XTL thickness profiler. The structural analysis of the synthesized films were performed by X-ray Diffractometer of model Bruker D8 Advance using monochromatic CuKα radiation (wavelength, *λ* = 1.5418 Å) in the range of 2*θ* = 20–80°. The Scanning Electron Microscope (SEM) of model ZEISS EVO-18 was used to visual analysis of surface topology of InSe films. The elemental composition of indium selenide thin films unrolled with an energy dispersive X-ray spectroscopy (EDS) detector attached with SEM. To understand chemical identification of synthesized indium selenide films FTIR spectra in the wavenumber range of 400–4000 cm^−1^ were also recorded. The FTIR spectra were monitored with a Shimadzu spectrometer (IRTracer-100). A T-60 ultraviolet-visible (UV-vis) Spectrophotometer (PG Instruments) was used to characterize optical properties by studying transmission spectra of indium selenide films.

## 3. Results and discussion

### 3.1 Annealing and stress effects on indium selenide thin films

Although annealing of films synthesized using thiol-amine cosolvents are performed in inert environment for avoiding oxidation, the synthesized indium selenide thin films were annealed in air. It was observed that annealing in air did not oxidize the films. The thickness of the synthesized films in both the unstressed and stressed cases was found to decrease with annealing temperature. The average thicknesses were ∼370, ∼360 and ∼330 nm for 250, 300 and 350 °C, respectively. However, the decrease in film thickness with the annealing temperature might occur due to the transformation of the film into more ordered phase with reduce defects that causes dimensional shrinkage resulting in the decrease of the film thickness.

**Fig. 2:**
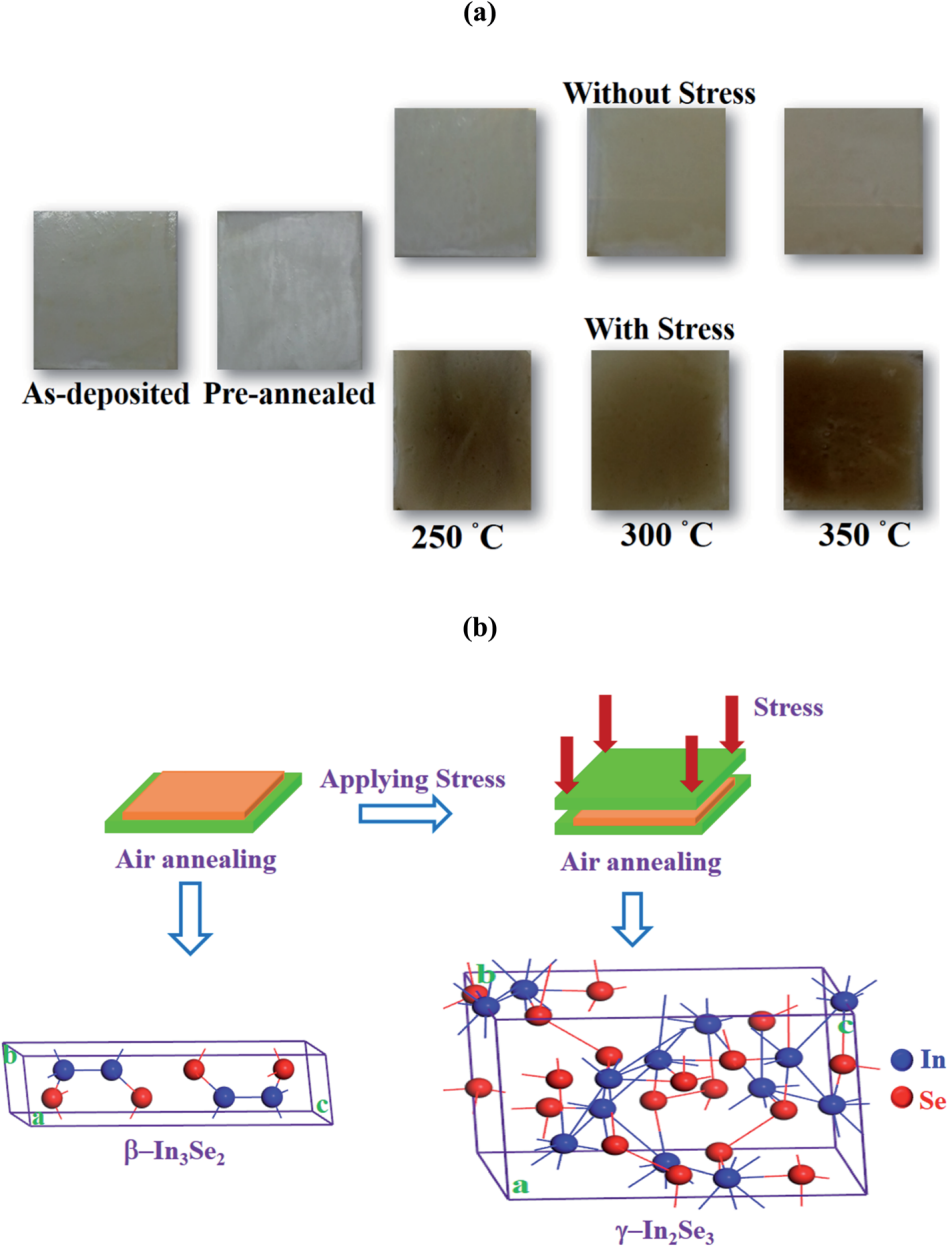
(a) The optical images of synthesized InSe thin films utilizing thiol-amine cosolvents at different annealing temperatures for without and with mechanical stress. (b) Schematic presentation of phase transformation of indium selenide thin films during annealing.

However, in a previous synthesis of CdS thin films through thiol-amine cosolvent route, a glass protector was used during annealing to suppress oxidation.^28^ The similar technique that provide simple stress on the films was utilized to visualize its effect on the films. Fig. 2a shows the optical images of indium selenide thin films deposited with and without stress using thiol-amine cosolvents. It is observed from figure that the films have turned into dark brown color from light brown due to the application of stress. This might be resulted from the phase alteration in indium selenide thin films from β-In_3_Se_2_ to γ-In_2_Se_3_ phase due to stress during annealing. The schematic of phase alteration of β-In_3_Se_2_ to γ-In_2_Se_3_ phase with their crystal structures are visualized in Fig. 2b. The β-In_3_Se_2_ phase has poly-hexagonal crystal structure with space group *P*6_3_/*mmc* and γ-In_2_Se_3_ crystal also has hexagonal structure with space group *P*6_1_ including distorted wurtzite-type atomic layout.

### 3.2 Structural analysis of indium selenide thin films

#### 3.2.1 XRD study of In_3_Se_2_ and In_2_Se_3_ thin films

**Fig. 3:**
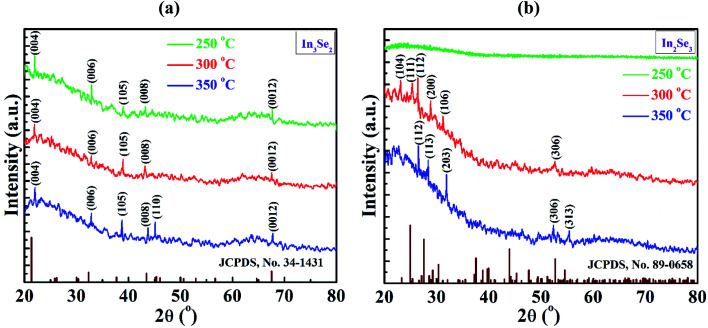
XRD spectra of indium selenide thin films for (a) In_3_Se_2_ and (b) In_2_Se_3_ phases, respectively deposited by spin coating method using thiol-amine cosolvents.

The XRD patterns of synthesized indium selenide thin films annealed at 250, 300 and 350 °C without and with stress are delineated in Fig. 3a and b, respectively. It is observed from Fig. 3a that the XRD peaks appeared at around 2*θ* = 21.9, 32.8, 38.7, 43.7, 45.15 and 67.7° for the films annealed without stress and these peaks can be indexed as (004), (006), (105), (008), (110) and (0012) planes, respectively (JCPDS card no. 34-1431) confirming the presence of polycrystalline hexagonal crystal structure of β-In_3_Se_2_ phase with the lattice parameters *a* = *b*= 4.005 and *c* = 16.640 Å.^12,29,30^

However, the phase is changed when a simple mechanical stress is applied on the films during annealing which is visualized in Fig. 3b. It is interesting to note that, there is no peak for the films annealed at 250 °C which indicates the amorphous nature of indium selenide thin films. The indium selenide thin films exhibited the same polycrystalline nature at annealing temperatures of 300 and 350 °C. For the films annealed at 300 °C, peaks were originated at around the angle 2*θ* = 23.16, 25.41, 26.42, 28.8, 31.26 and 52.6° which can be indexed as (104), (111), (112), (200), (106) and (306) planes, respectively. On the other hand, the films annealed at 350 °C exhibited five peaks which were also originated at around the angle 2*θ* = 26.54, 28.45, 31.95, 52.45 and 55.48° that can be indexed as (112), (113), (203), (306) and (313) planes, respectively according to JCPDS card no. 89-0658. This result indicates the presence of hexagonal structured γ-In_2_Se_3_ phase with lattice parameters *a* = *b*= 7.12 and *c* = 19.22 Å.^10,12,31^ It is also observed that with the presence of stress, the diffraction peaks become sharper with the increase of annealing temperature, suggesting a better crystallinity of γ-In_2_Se_3_ sample at 350 °C. It already is reported that γ-In_2_Se_3_ phase can be formed at a higher annealing temperature of 600 °C.^16^ However, in this study γ-In_2_Se_3_ phase in indium selenide films was formed at a low temperature of 300 °C and above which is mainly due to the application of mechanical stress. Hence, it can be concluded that solution-processed indium selenide thin films encounter the phase transformation from β-In_3_Se_2_ to γ-In_2_Se_3_ phase due to the application of mechanical stress during annealing in air.

However, it is also noticed from the figures that the crystallinity of the films in both the phases are relatively poor which could be improved with annealing at higher temperature. But, in this study, the high temperature annealing was not performed as this could result serious oxidation of the films as annealing was performed in open air.^28^ However, it is also true that very small amount of oxidation might be presented in amorphous form in the synthesized films.

The crystallite size *D*, full width half maximum (FWHM) *β*, the dislocation density *δ* were calculated from the XRD data to know crystallographic information. The crystallite size *D*, has been calculated using Debye–Scherrer's equation^28^1
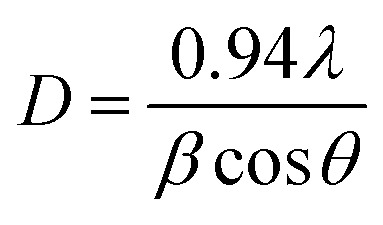
where, *λ* is the X-ray wavelength in nm, *β* is the FWHM in radians and *θ* is the Bragg angle of the involved peak.

The dislocation density, *δ* has also been estimated by using the formula^32^

2 *δ* = 1/*D*^2^

Table S1 in the ESI† shows the crystallographic parameters of β-In_3_Se_2_ and γ-In_2_Se_3_ phase of indium selenide thin films deposited by simple spin coating method using thiol-amine co-solvents. It can be seen from the table that the crystallite size of the β-In_3_Se_2_ phase varies in the range of 26–84 nm with dislocation density in the range of (1.4–14.5) × 10^14^ line per m^2^. Whereas the crystallite size of the γ-In_2_Se_3_ phase varies in the range of 24–56 nm with dislocation density in the range of (3.1–16.4) × 10^14^ line per m^2^. It is also observed that crystallite size decreases and dislocation density increases for the films annealed with stress. This lowering of the crystallite size and increase in dislocation density of the In_2_Se_3_ thin films due to the application of mechanical stress might be attributed to several factors such as differential stress, strain variation and grain size reduction, and the separation of the effect of each factor is difficult.^33^

#### 3.2.2 The SEM study of In_3_Se_2_ and In_2_Se_3_ thin films

**Fig. 4:**
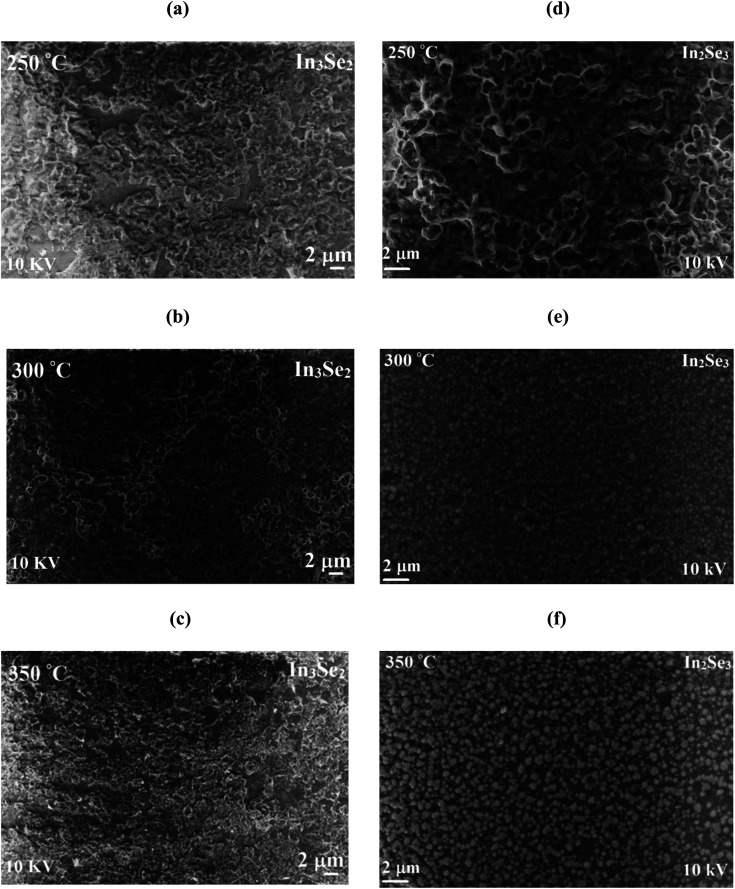
The SEM images of different magnifications of (a–c) In_3_Se_2_ and (d–f) In_2_Se_3_ phases, respectively of indium selenide thin films deposited by spin coating method using thiol-amine cosolvents.

Fig. 4a–c and d–f exhibit the SEM micrographs of fabricated In_3_Se_2_ and phase-transited In_2_Se_3_ thin films annealed at 250, 300 and 350 °C temperatures, respectively. It is observed from Fig. 4a–c that In_3_Se_2_ samples show the uniform surface morphology with slight shallow ditches. With the increase of annealing temperature, the fraction of ditches decreases owing to the pile of neighboring grains obtained from the thermal kinetic energy. The Fig. 4d–f assure that the resulting surface of In_2_Se_3_ thin fil–ms are uniform and compact but the surface roughness increases with annealing temperature. The agglomeration of the small grains spherical in shape is also detected with increase of annealing temperature. The In_2_Se_3_ thin films annealed at 300 °C provide better surface smoothness and uniformity compared to that annealed at 250 and 350 °C, respectively. It is also observed that the surface smoothness of In_2_Se_3_ phases are better and containing few ditches compared to In_3_Se_2_.

#### 3.2.3 The EDS study of indium selenide thin films

The EDS analysis was carried to evaluate the elemental composition of the synthesized indium selenide thin films. Fig. S1 in the ESI† presents the EDS spectra of In_3_Se_2_ and In_2_Se_3_ phases, respectively of indium selenide thin films. The atomic percentages of constituent elements for both phases are shown in Table 1. It is seen from table that In and Se ratio is very close to 3 : 2 for the films annealed without stress at temperatures of 250, 300 and 350 °C, respectively. This indicate the stoichiometric In_3_Se_2_ phase which is consistent with the reported work.^29^ However, the starting materials for the solution was InSe with 1 : 1 ratio which ended up with 3 : 2 ratio of In and Se in In_3_Se_2_ thin films. This may be happened due to the re-evaporation of Se during annealing which agreed well with the other reports.^4,34^ On the other hand, In and Se ratio is nearly 2 : 3 for the films annealed with stress at annealing temperatures of 250, 300 and 350 °C, respectively which ensures the stoichiometric In_2_Se_3_ phase of the films.^35,36^ Here, the thin films are synthesized with 2 : 3 ratio of In and Se, respectively. This may be happened due to the re-arrangement of In and Se atoms and/or re-evaporation of In during annealing and this result is also consistent with the reported works.^12,37^ For the both cases, it is also observed from table that the stoichiometric ratio increases with increasing annealing temperatures. The EDS mapping for γ-In_2_Se_3_ phase is also shown Fig. S2 in ESI,† which also indicates the increase of In and decrease of Se with increasing annealing temperatures. In the EDS spectra, another component Si (not shown) was detected which was mainly originated from the glass substrate. Other few components such as Na, Mg, O, N, Pt, Ca, S and C were detected in negligibly small amount that were also ignored in EDS spectra. Therefore, the phase transformation from In_3_Se_2_ to In_2_Se_3_ phase due to stress during annealing can also be assured from EDS study.

**Table 1:** Elemental compositions of solution-processed InSe thin films deposited without and with a mechanical stress

Annealing temperature (°C)	In_3_Se_2_ phase			In_2_Se_3_ phase		
	Elements		Stoichiometric ratio In/Se	Elements		Stoichiometric ratio In/Se
	In (at%)	Se (at%)		In (at%)	Se (at%)	
250	56.92	43.08	1.32	37.25	62.75	0.594
300	61.03	38.97	1.57	40.10	59.90	0.669
350	62.32	37.68	1.65	41.40	58.60	0.706

#### 3.2.4 The FTIR analysis of indium selenide thin films

**Fig. 5:**
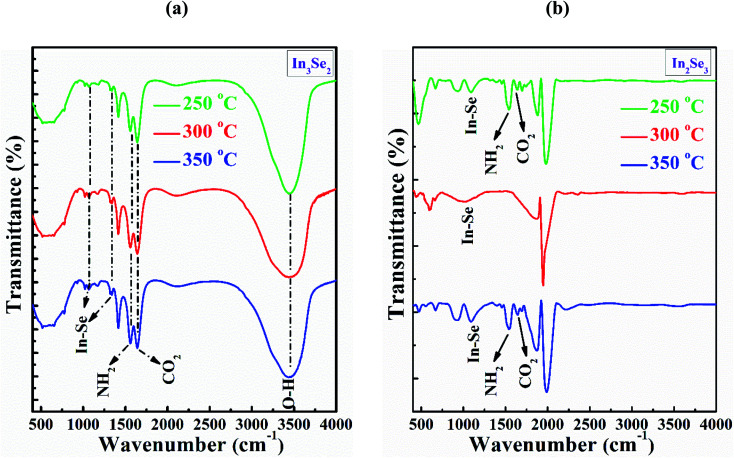
The FTIR spectra of InSe thin films for (a) In_3_Se_2_ and (b) In_2_Se_3_ phase, respectively deposited by spin coating method using thiol-amine cosolvents.

The FTIR spectra were recorded in the range of 400 to 4000 cm^−1^ in order to explore chemical bonding of the synthesized In_3_Se_2_ and In_2_Se_3_ thin films annealed at 250, 300 and 350 °C as depicted in Fig. 5a and b, respectively. The peak intensity in the range of absorption band at 1000 and 1300 cm^−1^ wavenumber indicates the different phases of In–Se stretching vibration bonds.^38^ In Fig. 5a, two peaks corresponds to In–Se stretching vibration bonds are found at the absorption band at 1067 cm^−1^ and 1340 cm^−1^ of In_3_Se_2_ phase. Another absorption peak at band at 1560 cm^−1^ indicates NH_2_ scissor vibrations bond which may be originated from cosolvents ethylene di-amine.^39^ The other absorption peak at 1640 cm^−1^ wavenumber is attributed to CO_2_ molecule which may grow due to the air environment.^38,40^ The peak at ∼3435 cm^−1^ for In_3_Se_2_ thin films represents the fundamental stretching vibration of O–H bond of hydroxide group or water moisture adsorbed at the surfaces, while this peak is absent in case of In_2_Se_3_ thin films. This result might be due to the more possible access of air, which causes air oxidation of In_3_Se_2_ to the surface of the thin film. On the other hand, the covering of the films by glass protector (stressed) during annealing might restrict the access of air oxygen to some extent resulting the O–H free thin films.^41^

Moreover, the peak intensity at the absorption band at 1090 cm^−1^ is found for the films annealed at 250 and 350 °C, respectively suggest the In–Se stretching vibration bonds of the of In_2_Se_3_ phase as shown in Fig. 5b. The small peaks at the absorption band at 1640 cm^−1^ are originated for CO_2_ molecule similar to In_3_Se_2_ phase.^38,40^ Another peak at the wavenumber 1022 cm^−1^ is also responsible for In–Se stretching vibration bond as appeared for the film annealed at 300 °C. The NH_2_ scissor vibrations bond is also identified at the absorption band at 1560 cm^−1^ for the annealing with stress which has come from cosolvents ethylene di-amine.^39^

### 3.3 The electrical properties of indium selenide thin films

**Fig. 6:**
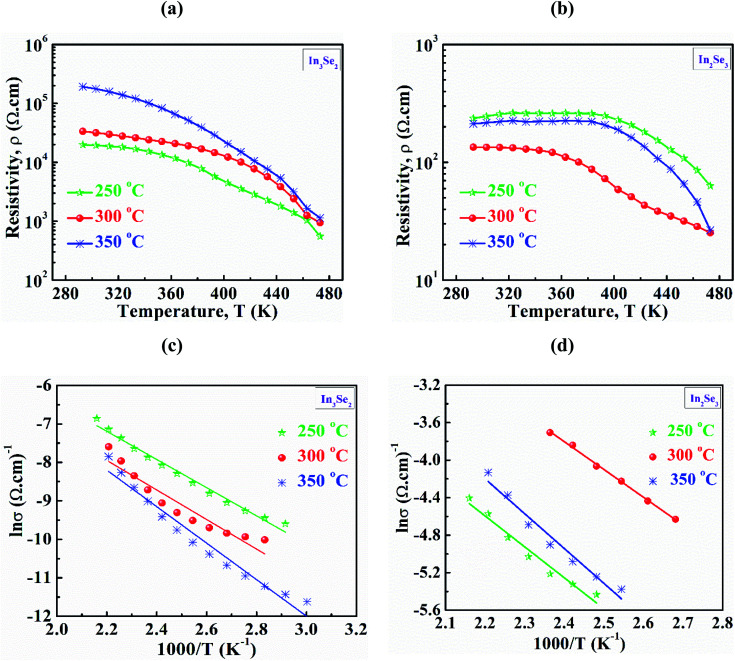
The temperature dependent electrical resistivity (a and b), variation of ln *σ vs. T*^−1^ of In_3_Se_2_ and In_2_Se_3_ thin films (c and d), respectively deposited by spin coating method using thiol-amine cosolvents.

Temperature dependent resistivity (*ρ*) of synthesized In_3_Se_2_ and In_2_Se_3_ thin films in the temperature range of 293–473 K are presented in Fig. 6a and b, respectively. Fig. S3 (a and b) in ESI† also presents temperature dependent conductivity (*σ*) in the range of 293–473 K of In_3_Se_2_ and In_2_Se_3_ thin films, respectively. It is noticed that for all samples the resistivity decreases with temperature which indicates the semiconducting nature of the films.^4^ It can also be seen that resistivity decreases significantly due to the application of stress in the samples during annealing. For the case of In_3_Se_2_ phase, the room temperature resistivity was in the magnitude of 10^4^ (Ω cm) which decreased to 10^3^ (Ω cm) at a temperature of 473 K for the films annealed at 250 and 300 °C, respectively as shown in Fig. 6a. However, the resistivity was in the order of 10^5^ (Ω cm) for the films annealed at 350 °C and decreased to the order of 10^3^ (Ω cm) at a temperature of 473 K. This difference in resistivity may be related to the surface morphology of the films as SEM images show that the In_3_Se_2_ films annealed at 350 °C is more discontinuous. On the other hand, the room temperature resistivity of In_2_Se_3_ phase was found in the order of 10^2^ (Ω cm) for all annealing conditions and it decreased to the order of 10^1^ (Ω cm) at a temperature of 473 K as shown in Fig. 6b. The In_2_Se_3_ thin films annealed at 300 °C exhibited less resistivity. This reduction of resistivity may also be associated with the surface morphology as SEM images show that the film annealed at 300 °C is relatively smooth and uniform compared to other samples leading to less carrier scattering effect.

Fig. 6c and d depict the plots of ln *σ vs. T*^−1^ of In_3_Se_2_ and In_2_Se_3_ thin films, respectively for different annealed temperatures. The slopes of ln *σ vs. T*^−1^ plots are used to estimate the activation energies of In_3_Se_2_ and In_2_Se_3_ thin films using the following eqn (3)^42^3
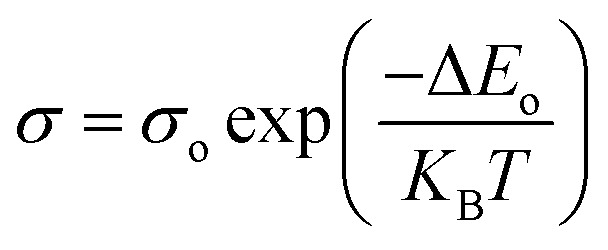
where, Δ*E*_o_ is the activation energy, *σ* is the conductivity, *σ*_o_ is the pre-exponential factor and *K*_B_ is the Boltzmann constant. The estimated activation energy and pre-exponential factor are shown in Table 2. Activation energies are found in the range of 0.32–0.40 eV and 0.26–0.32 eV for In_3_Se_2_ and In_2_Se_3_ phases, respectively which are in good agreement with previous works.^43,44^ As seen on the table, the value of activation energies, Δ*E*_o_ > 3*K*_B_*T* which also indicate that these samples are non-degenerate semiconductors.^42^ It is also seen from the table that the pre-exponential factor is higher for the films with In_2_Se_3_ phase. The pre-exponential factor might be associated with the ion diffusion and nuclei surface properties.^45^ The nucleation rate further depends on temperature. XRD results show that average crystallite size of In_2_Se_3_ phase reduces after inducing stress. Likewise, as observed in SEM micrographs, nucleation rate of In_2_Se_3_ sample is more significant than that of In_3_Se_2_ phase and the number of nuclei is appear to increase with increasing temperature. In this work, the increase of the pre-exponential factor after phase transition may be attributed to the increase of nuclei diffusion rate. However, further investigation is needed to find out details of the exact reasons of this phenomenon.

**Table 2:** Calculated activation energy, Δ*E*_o_ and pre-exponential factor, *σ*_o_ for In_3_Se_2_ and In_2_Se_3_ phases of indium selenide thin films

Annealing temperature (°C)	In_3_Se_2_ phase		In_2_Se_3_ phase	
	Activation energy, Δ*E*_o_ (eV)	Pre-exponential factor, *σ*_o_ (Ω cm)^−1^	Activation energy, Δ*E*_o_ (eV)	Pre-exponential factor, *σ*_o_ (Ω cm)^−1^
250	0.32	2.36	0.29	14.16
300	0.33	1.76	0.26	27.38
350	0.40	9.30	0.32	52.98

### 3.4 The optical properties of indium selenide thin films

**Fig. 7:**
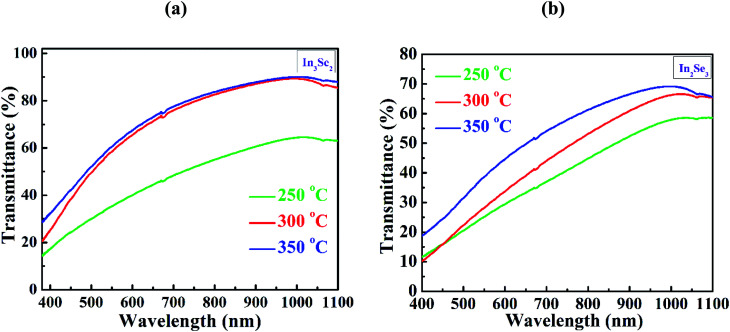
The optical transmittance spectra of spin coated (a) In_3_Se_2_ and (b) In_2_Se_3_ thin films, respectively prepared using thiol-amine co-solvents.

The optical transmittance in the range of photon wavelength 360–1100 nm of spin coated In_3_Se_2_ and In_2_Se_3_ thin films for different annealing temperatures was measured as exhibited in Fig. 7a and b, respectively. It is observed from the figures that for both phases transmittance increases with increasing annealing temperature. When the samples are annealed at 350 °C, the β-In_3_Se_2_ phase of indium selenide thin films exhibit maximum transmittance which is significantly higher than the transmittance of the γ-In_2_Se_3_ samples. The transmittance of the β-In_3_Se_2_ samples with increasing annealing temperature can be correlated with the crystallinity and surface morphology of the films. As seen in XRD and SEM, better crystallinity and higher surface discontinuity was resulted for the sample annealed at 350 °C. Besides, the transmittance of the In_2_Se_3_ thin films also increases with annealing temperature which might be resulted due to the better crystallinity of the films with annealing temperature as shown in Fig. 7b. It can also be seen that for the both phases the transmittance decreases continuously with wavelength in the visible region from infrared region. This may happened due to the increasing of scattering loss in the visible region.^34^ It is also observed from the both figures that at wavelength around 676 nm there is a small unwanted bump which was generated due to instrumental error.^46^ The absorption spectra for both phases are shown in Fig. S4 in ESI.†

The absorption coefficient, *α* was computed from the transmittance spectra using the following eqn (4).^28^4
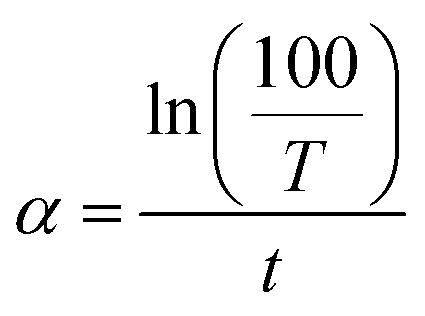
where, *T* is the transmittance and *t* is the thickness of thin films.

The absorption coefficient was found in the order of 10^4^ cm^−1^ for both phases which is also similar to earlier report.^34^

The absorption coefficient (*α*) and photon energy (*hν*) are related with the Tauc equation as^47^

5 (*αhν*)^2^ = *A*(*hν* − *E*_g_)

where, *E*_g_ is the optical band gap and *A* is the constant. The extrapolation of the linear portion of (*αhν*)^2^*versus* photon energy (*hν*) plot provides energy band gap *E*_g_ = *hν* when (*αhν*)^2^ = 0.

**Fig. 8:**
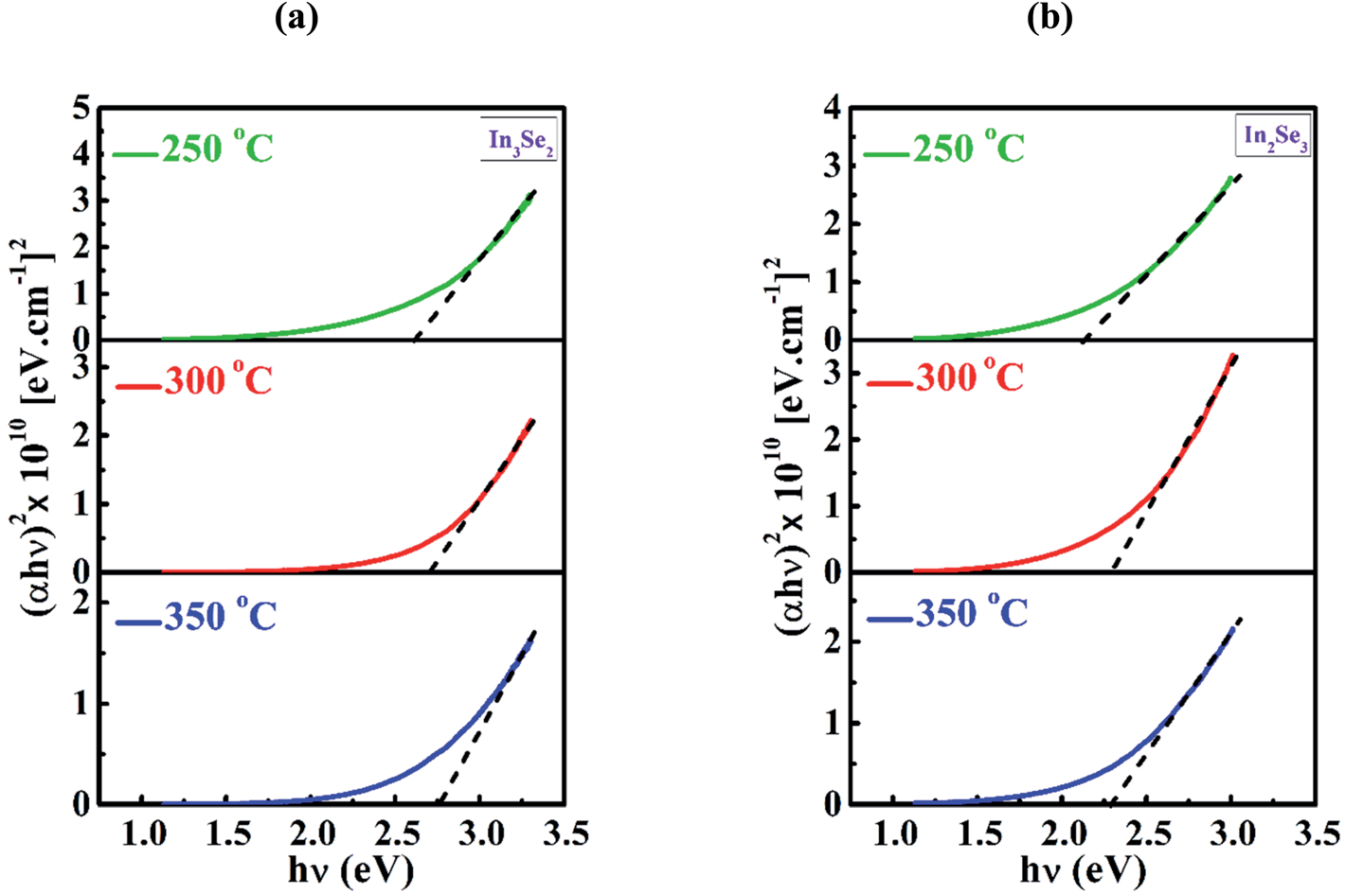
The Tauc plots of (a) In_3_Se_2_ and (b) In_2_Se_3_ phases of indium selenide thin films, respectively prepared using thiol-amine co-solvents showing the band gap of the films annealed at different temperatures.

Fig. 8a and b represent the Tauc plots of deposited In_3_Se_2_ and In_2_Se_3_ phases of indium selenide thin films, respectively for different annealing temperatures. In this work, the direct band gap of In_3_Se_2_ phases were found to be 2.60, 2.70 and 2.75 eV and that for In_2_Se_3_ phase were found to be 2.11 eV, 2.28 eV and 2.28 eV at annealing temperatures of 250, 300 and 350 °C, respectively. It is observed from the figures that band gap of both the phases increases with annealing temperatures. This increase in optical band gap with annealing temperature might occurred owing to the reduction of the disorder and defects in the structural bonding that were presented in the films. As the microstructural modifications in a thin film material rely on the growth kinetics and the substrate temperature, the uncompleted microstructures might be changed due to thermal energy of post-depositional annealing temperatures. The increase of optical band gap of indium selenide thin films due to annealing has already been reported.^19,37,47^ However, there are also few reports on nanostructured perovskite semiconductors indicating that band gap can be decreased through phase transformation and quantum confinement.^48,49^ The high band gap for In_3_Se_2_ phase at various annealing temperatures can be found in other reports.^50,51^ The band gap of In_2_Se_3_ phase is also agreed well with the reported values.^52^

## 4. Conclusions

The indium selenide thin films have been synthesized by spin coating method using InSe solution in thiol-amine cosolvents. The films encounter phase transformation from β-In_3_Se_2_ to γ-In_2_Se_3_ phase due to a mechanical stress applied by 1–1.2 mm thick microscopic glass pressed with crocodile clips during annealing. The XRD study revealed the β-In_3_Se_2_ and γ-In_2_Se_3_ phases of synthesized indium selenide thin films without and with stress, respectively. The smooth and uniform surface were demonstrated by SEM micrographs for both phases. The EDS study showed the stoichiometric In_3_Se_2_ and In_2_Se_3_ phases. The In–Se stretching vibration bonds were noticed by FTIR study. Temperature dependent electrical study also revealed the non-degenerate indium selenide thin films for both phases. The transmittance spectra showed the maximum transmittance of approximately 77.5 and 54% at a wavelength of 700 nm for β-In_3_Se_2_ and γ-In_2_Se_3_ phases, respectively at an annealed temperature 350 °C. The absorption coefficient was found in the order of 10^4^ cm^1^. The optical band gap was also calculated in the range of 2.60–2.75 and 2.12–2.28 eV for β-In_3_Se_2_ and γ-In_2_Se_3_ phases, respectively. These findings suggest that phase change in solution processed indium selenide might be applied in 2D optoelectronic devices in near future.

## Conflicts of interest

The authors declare no conflict of interests.

## Supplementary Material

RA-011-D1RA01403J-s001
