# Supplementary material for: Stress-induced phase-alteration in solution processed indium selenide thin films during annealing
Source: RSC Adv. 2021 Apr 13;11(23):13751–62. doi: 10.1039/d1ra01403j (PMC8697694; doi:10.1039/d1ra01403j)
Supplement: RA-011-D1RA01403J-s001 [file RA-011-D1RA01403J-s001.pdf]

Electronic Supplementary Material (ESI) for RSC Advances.
This journal is © The Royal Society of Chemistry 2021

Supporting Information

Stress-induced phase-alteration in solution processed indium selenide thin films during annealing

Bipanko Kumar Mondal[1], Shaikh Khaled Mostaque[1], Md. Ariful Islam[2] and Jaker Hossain[1]*

[1]*Solar Energy Laboratory, Department of Electrical and Electronic Engineering, University of Rajshahi, Rajshahi 6205, Bangladesh.*

[2]*Department of Physics, Rajshahi University of Engineering and Technology, Rajshahi 6204, Bangladesh.*

Corresponding Authors

*E-mail: jak_apee@ru.ac.bd

Table S1: Crystallite size and Dislocation densities of synthesized In_3Se_2 and In_2Se_3 thin films.

**Phase**	**Annealing temperature (°C)**	**Planes (hkl)**	**FWHM, β (Rad) × 10^{-3}**	**Crystallite Size, D (nm)**	**Dislocation density, δ (lin/m^2) × 10^{14}**
In_3Se_2	250	004	1.75	84.58	1.40
		006	2.44	61.83	2.62
		105	3.49	44.04	5.16
		008	3.90	40.60	6.07
		0012	3.14	55.52	3.24
	300	004	1.75	84.58	1.40
		006	3.14	48.09	4.33
		105	5.06	30.37	10.85
		008	5.94	26.27	14.49
		0012	3.14	55.52	3.24
	350	004	2.96	49.75	4.04
		006	3.50	43.28	5.34
		105	3.14	48.90	4.18
		008	4.36	35.79	7.81
		110	2.44	64.24	2.42
		0012	3.14	55.55	3.24
In_2Se_3	300	104	4.02	36.85	7.36
		111	4.02	37.01	7.30
		112	2.62	56.86	3.09
		200	2.97	50.43	3.93
		106	3.49	43.12	5.38
		306	4.89	33.08	9.14
	350	112	2.62	56.88	3.09
		113	5.59	26.77	14.0
		203	2.79	53.98	3.43
		306	4.71	34.29	8.50
		313	6.63	24.69	16.40

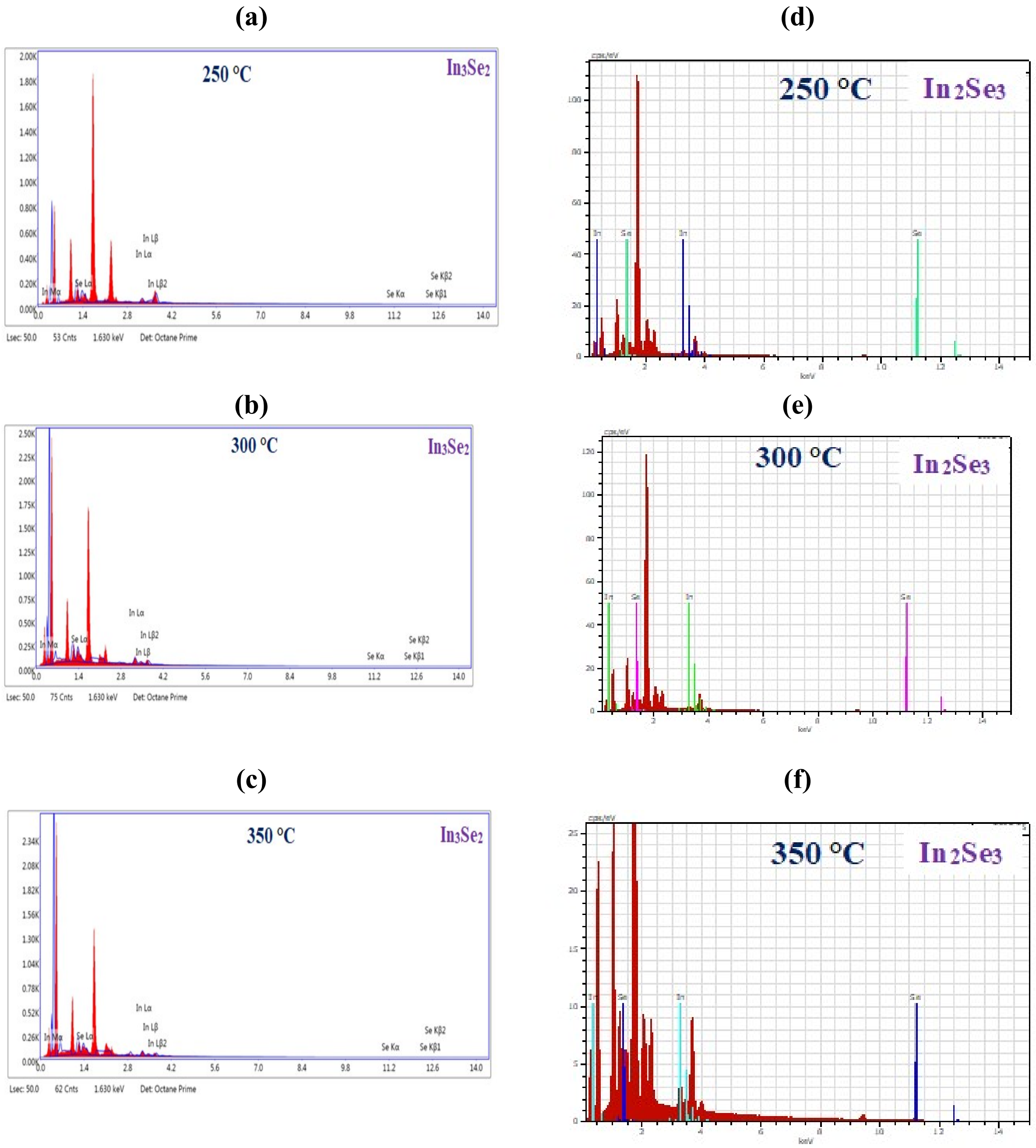

Fig. S1. The EDS spectra of In_3Se_2 and In_2Se_3 thin film deposited by spin coating method using thiol-amine cosolvents.

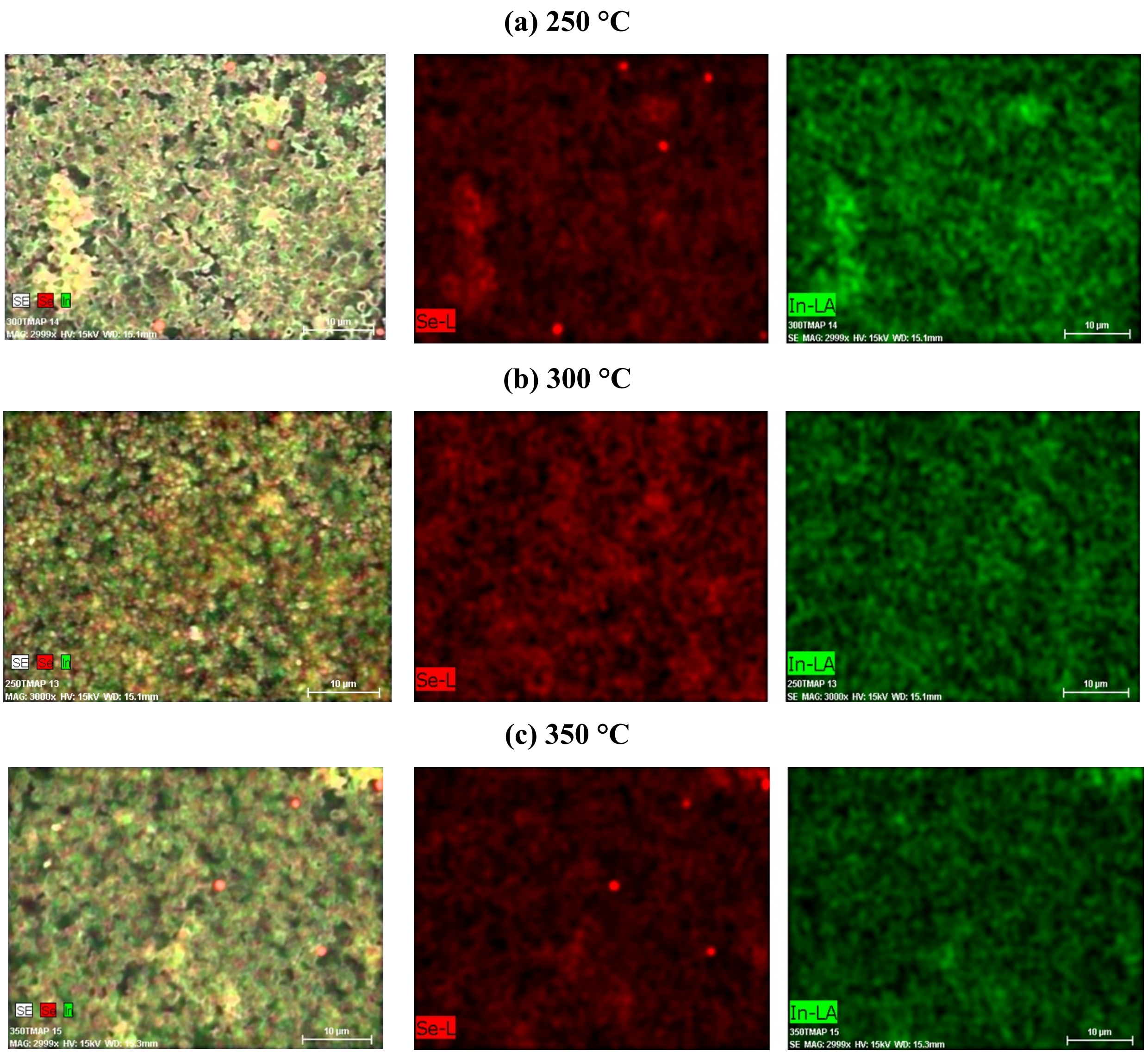

Fig. S2. EDS mapping of In_2Se_3 thin films deposited by spin coating method using thiol-amine cosolvents.

(a) **(b)**

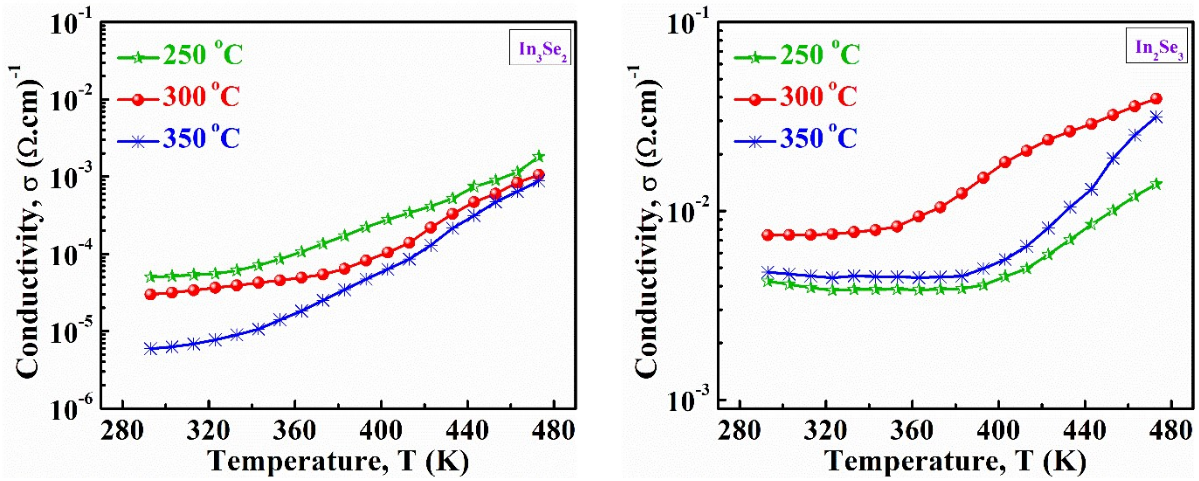

Fig. S3. The electrical study (a-b) temperature dependent conductivity of In_3Se_2 and In_2Se_3 thin films, respectively deposited by spin coating method using thiol-amine cosolvents.

(a) **(b)**

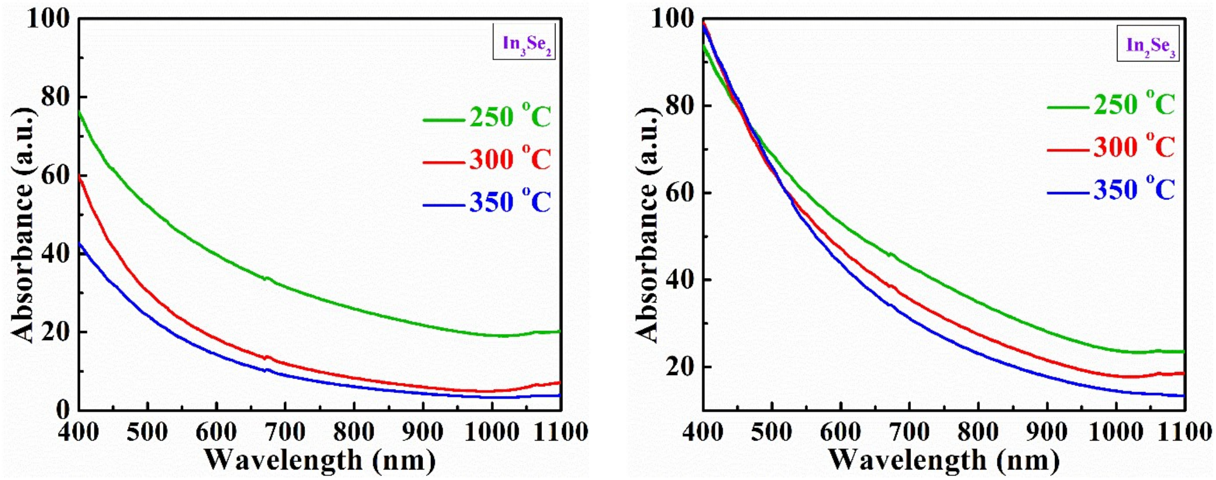

Fig. S4. The (a-b) optical absorbance spectra of spin coated In_3Se_2 and In_2Se_3 thin films, respectively prepared using thiol-amine co-solvents
